# Isolation of Potential Phages against Multidrug-Resistant Bacterial Isolates: Promising Agents in the Rivers of Kathmandu, Nepal

**DOI:** 10.1155/2017/3723254

**Published:** 2017-11-22

**Authors:** Anjeela Bhetwal, Anjila Maharjan, Shreena Shakya, Deepa Satyal, Sumitra Ghimire, Puspa Raj Khanal, Narayan Prasad Parajuli

**Affiliations:** ^1^Department of Laboratory Medicine, Manmohan Memorial Institute of Health Sciences, Kathmandu, Nepal; ^2^Department of Clinical Laboratory Services, Manmohan Memorial Medical College and Teaching Hospital, Kathmandu, Nepal

## Abstract

Bacteriophages are being the subject of interest for alternative antimicrobial therapy for infectious diseases in recent years. Therapeutic effectiveness regarding phage therapy is a matter of concern since it is the most promising biological treatment of this era. Hence, the present study was aimed to isolate the potential bacteriophages present in river water samples and to analyze their host range among clinical strains of bacteria. Ten different locations of Kathmandu valley were selected for the collection of river water for the detection of probable phages. Bacteriophages were isolated from water samples using the double agar overlay method. Isolated phages were purified by diluting in the SM-buffer and filtering through 0.22 *μ*m filter. Purified lysate was further processed for analyzing its host range by using spot method. Their host range was characterized against 20 bacterial strains, including multidrug-resistant. Total 67 different phages were isolated against 8 different host organisms. Out of them, forty-seven phages were selected for analyzing its host range. Among them,* Serratia *phages (ΦSER) had the broad host range infecting 17 different bacterial strains including multidrug-resistant harboring ESBL and MBL genotypes. However,* Klebsiella *phages (ΦKP) had narrow host range in comparison to other phages. Isolated phages had the potential effect against clinical strains of bacteria along with their broader host spectrum. Most importantly, promising effect against MDR pathogens in this study has raised the probable chances of the utility of these phages for biological control of bacterial infection including MBL and ESBL strains.

## 1. Background

Globally, dissemination of multidrug resistance among bacterial strains has posed a significant threat to public health confronting the routine treatment of infectious diseases [1, 2]. Despite the global surge of such resistant bugs, development of new antibiotics has been decelerated since last few decades [3]. Therefore, it necessitates the incessant endeavors to develop a promising alternative for treating infectious diseases and reducing the emergence and dissemination of antibiotic resistance among pathogens [4, 5]. Recently, bacteriophages are gaining new ground as an alternative regime for the therapeutic application as they impose antibacterial properties and self-replicate during infection [1, 6]. Hence, there is renaissance in the use of bacteriophages to counteract the resistant pathogens [7].

Bacteriophages (“phages” for short) possess novel mode of action compared to that of antibacterial regimens, as they selectively infect pathogenic bacteria including multidrug-resistant pathogens (in vivo and in vitro) [8]. Furthermore, they are ecologically safe and effective in lower doses and do not show adverse reactions on their application in human body [1, 9]. To these assets, phages have garnered increasing attention in the therapeutic application in recent years. Several studies, available to date, have revealed the lytic efficacy of phages against various pathogenic organisms including* Escherichia coli, Klebsiella pneumoniae, Pseudomonas aeruginosa, Acinetobacter baumannii, Vibrio cholerae, Salmonella species, Staphylococcus aureus, Enterococcus *spp., and* Serratia *spp. [10–15]. In addition, ability of lytic phages against multidrug-resistant bacteria producing hydrolytic enzymes including extended spectrum *β*-lactamases (ESBL) producing* E. coli*,* Pseudomonas aeruginosa*,* Klebsiella pneumoniae*, Methicillin-Resistant* Staphylococcus aureus *(MRSA), and Vancomycin-resistant* Enterococcus* has also been reported [16–19]. These findings are extremely important for application of phages in the treatment of infectious diseases associated with resistant bugs. From the very beginning of their discovery, phages have been used for treating various bacterial infections in some developed countries of Europe [20]. Although there were initial few experiments, research on “phage therapy” was declined in the West and United States, but looming antibiotic crisis has renewed interest in the extensive use of phages in recent years [21].

In Nepal, there is continuous increment of antimicrobial resistance among pathogenic bacterial strains. Despite the growing menace of antimicrobial resistance in our country, there is very little attention being paid for its control and newer alternatives have not been investigated yet. Alongside, as a well-off country in water resources with plenty of rivers, investigation of lytic phages in our holy rivers could be a promising alternative to overcome the effect of antimicrobial resistance. However, there is no such previous study documenting the isolation of phages from Nepalese rivers and analysis of these phages against drug-resistant bacterial isolates. In this backdrop, we have tried to isolate various potential phages against pathogenic bacteria including multidrug-resistant strains and to explore their potential host range among bacterial isolates.

## 2. Materials and Methods

### 2.1. Study Design

A descriptive cross-sectional study was carried out in the Department of Microbiology, Manmohan Memorial Institute of Health Sciences, Kathmandu, for the analysis of potent phages present in water samples from various rivers of the Kathmandu valley and their lytic effect on pathogenic bacterial strains. Over the period of six months, ten different sources of phages were identified and extensive purification and analysis of their effect on pathogenic bacteria were investigated. Approval from Kathmandu Metropolitan City was obtained before collecting the specimens from river.

### 2.2. Water Specimens

Total ten river water samples were collected from different locations of Kathmandu valley. Samples were collected from stagnant surface of river in a 100 ml sterile glass bottle. After removal of larger particulates by centrifugation at 3000 rpm for 30 minutes, the supernatant was slowly filtered through a syringe filter (Whatman 25 mm GD/X) with a pore size of 0.22 *μ*m to a sterile 15 ml screw capped tube (Borosil).

### 2.3. Bacterial Strains

Bacterial isolates from various clinical specimens from patients (sputum, blood, pus, urine, and other body fluids) were isolated and identified by standard microbiological methods suggested by American Society for Microbiology (ASM) [22]. Only the clinical strains were used as there is unavailability of commercial bacterial strains in Nepal. Antimicrobial susceptibility against different antibiotics was tested by the disk diffusion method (modified Kirby-Bauer) on Mueller-Hinton Agar (HiMedia Laboratories, India) as recommended by Clinical and Laboratory Standards Institute (CLSI) [23]. For instance, strains that are resistant to at least one agent in three classes of first-line antimicrobial agent were considered as multidrug-resistant (MDR) organism [24]. Extended spectrum *β*-lactamase (ESBL) encoding genes of the family Temoniera (TEM), sulfhydryl variable (SHV), and Cefotaximase-Munich (CTX-M) were detected by polymerase chain reaction using specific primers [25]. Metallo-*β*-lactamase (MBL) producing organisms were detected using combined disk method (i.e., Imipenem and Imipenem + EDTA) as suggested by Yong et al. [26]. These isolates were used for analysis of the effectiveness of various phages. Total eight different types of isolates including* Escherichia coli*,* Klebsiella pneumoniae, Citrobacter koseri, Enterobacter cloacae, Proteus mirabilis, Pseudomonas aeruginosa, Serratia marcescens*, and* Salmonella typhi *were used as a host strains for phage isolation. Briefly, two to three colonies of these organisms were emulsified with peptone water and incubated for a period of 4 hours at 37°C to adjust the inoculum density equal to that of 0.5 MacFarland turbidity standards.

### 2.4. Bacteriophage Isolation (Plaque Assay)

One milliliter of phage filtrate was transferred into a sterile tube. Then, 50 *μ*l of the respective host suspension was added and mixed well. It was left for 10 minutes at ambient temperature for allowing phage to adsorb to the host. After 10 minutes, 3 ml of 0.7% molten agar (at 50°C) was added, mixed well, and poured over the surface of nutrient agar plate. It was allowed to set at room temperature and incubated at 37°C for 24 hours. Plates were observed and scored positive if there was a presence of clear zone (plaque formation) over the surface of the agar plate. Plaques were counted from all positive samples and recorded as a plaque forming unit (pfu/ml) [27].

### 2.5. Purification of Phages

For purification of the phages, clear plaques were selected and plugged off from the agar surface using sterile pipette tips and then mixed in 10 ml SM-buffer with agitation in vortex mixer. The agar and cell residues were removed by centrifugation at 3000 rpm for 30 min, followed by filtration of the supernatant through a 0.22 *μ*m pore sized syringe filter. Resulting filtrate (phage lysate) was preserved at 4°C until processing [27].

### 2.6. Determination of Host Range

The host range of isolated phages was determined by spot test using 20 different bacterial strains. The plate was marked to allow identification of each phage. A sterile cotton swab was moistened with the broth culture and lawn culture was made on the surface of nutrient agar (HiMedia Laboratories, India) plate from each bacterial strain. Five microliters (5 *μ*l) of each phage lysate was spotted on the marked area of the agar plate. Lysates were allowed to dry before incubation at 37°C for 24 hours. Plates were observed for lytic zone formed on the spotted area and the effectiveness of individual phage was noted [28].

## 3. Results

### 3.1. Spectrum of Phages

**Figure 1:**
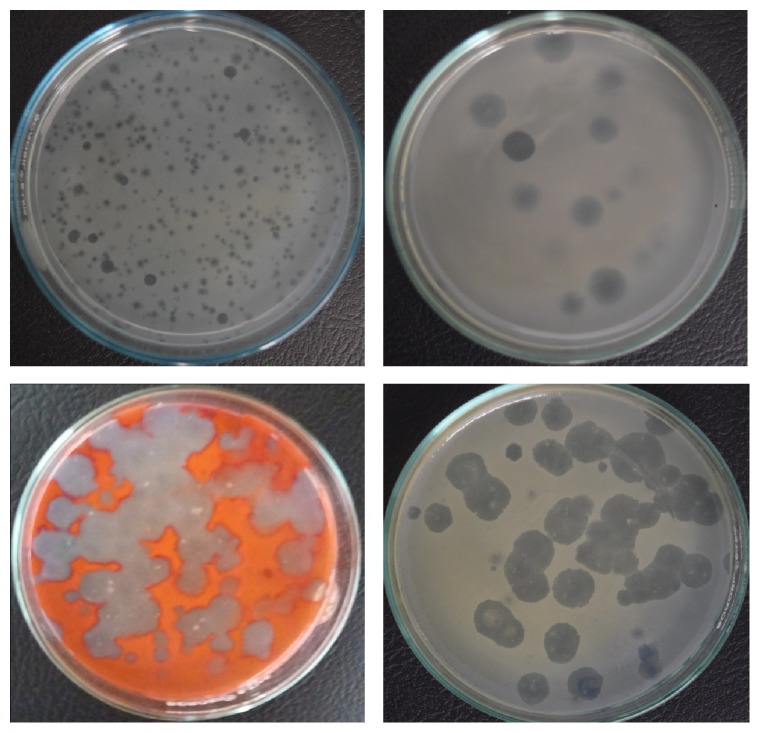
Potential phages against* Citrobacter *spp.*, Proteus *spp.*, Serratia *spp.,* and Klebsiella* spp.

Total ten water samples were screened for the presence of phages. Using 8 different bacterial strains as host organisms, 67 phages were isolated from ten river water samples by double agar overlay method. All the samples yield phage against bacterial isolates including* Escherichia coli, Klebsiella pneumoniae, Enterobacter cloacae, Serratia marcescens*, and* Citrobacter koseri*. Likewise, 8 water samples were found to contain phages against* Proteus*, 5 samples against* Pseudomonas*, and 4 samples against* Salmonella*. These phages produced clear and turbid plaques of different sizes (Figure 1). Isolated phages were named according to the bacterial species and sample number (e.g., phage ΦEC1 stands for host organism* Escherichia coli* and sample number 1 from where it was isolated). Spectrum of effective phages and numbers of plaques produced against individual host are illustrated in Table 1.

### 3.2. Host Range of the Isolated Phages

**Figure 2:**
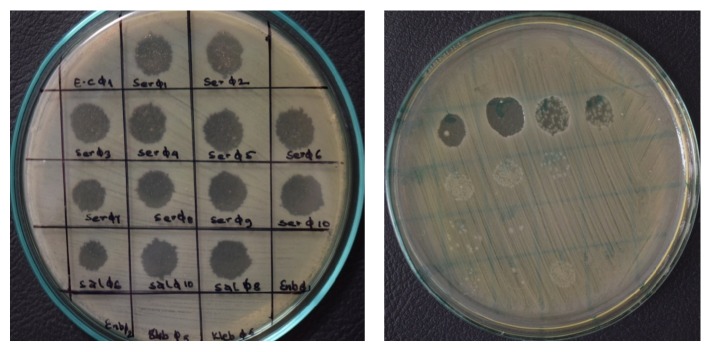
Analysis of host range for isolated phages.

Phages were selected on the basis of the size and clarity of plaques they produced for screening their host range. Thus, the infectivity of forty-seven phages was analyzed against 20 different bacterial isolates including genetically characterized MDR strains. Host range of these phages was investigated by spot method which revealed that majority of phages were able to lyse pathogenic strains. Their infectivity was categorized on the basis of plaque size and its intensity. The plaques were categorized as very effective (+ + + +), fairly effective (+ + +), moderately effective (+ +), and slightly effective (+) based on the degree of their clarity (Figure 2).

Among 47 phages, ΦSER1 was the most effective phage with 85% lytic ability killing 17 different bacterial strains. The intensity of this phage was fairly effective (+ + +) to* Escherichia coli*,* Klebsiella pneumoniae*,* Enterobacter *spp. and* Pseudomonas *spp., while being moderately effective (+ +) to MDR* Pseudomonas *spp. and slightly effective (+) to* Citrobacter koseri* indicating broad host range, while* Proteus* and* Salmonella* were resistant to this phage. Moreover, this phage was also found to be effective against multidrug-resistant isolates like* Klebsiella* spp. and* Escherichia coli* harboring bla-SHV, bla-TEM, and bla CTX-M and bla-SHV + bla-CTX-M genes.

On the other hand, phages isolated from* Klebsiella* spp., ΦKP6, ΦKP7, ΦKP8, and ΦKP9, were found least effective (5% lytic effect). Thus ΦKP6, ΦKP7, ΦKP8, and ΦKP9 seem to be specific only to* Klebsiella pneumoniae*. The information about lytic ability of various phages from bacterial isolates and their host range is illustrated in Tables 2, 3, 4, 5, and 6.

## 4. Discussion

Resistant pathogens are ever increasing and it is anticipated that those pathogens would emerge as a substantial global problem. These emerging MDR pathogens and unavailability of newer antibiotics has reintroduced the use of phages cited to its specificity and novel mode of action. Hence, treatment of these menacing pathogens with the lytic bacteriophage and researches on it is gaining spotlight in this era [29]. To the best of our knowledge, this is probably the first study conducted in Nepal, particularly on isolation and characterization of the phages.

Bacteriophages are ubiquitous in the environment where their host resides such as rivers, soil, sewage, poultry or animal feces, water ponds, and sea water [30]. In general, river water contains large diversity of enteric organisms due to fecal contamination of rivers in our country. The present study attempted to isolate phages from river water samples as they are the most relevant sources for its isolation. Moreover, in other studies, isolation of phages from fresh water ponds, animal wastes, and soil was successful too. Shukla et al. (2014) isolated phages in animal waste collected from different livestock's farming [31]. Likewise, the study carried out by Li and Zhang (2014) isolated phage specific against* Staphylococcus aureus* by processing fresh milk samples collected from local dairy farm [32]. Similarly, Alonso et al. isolated 26 phages from water samples of Alboran Sea [33]. Seaman and Day (2007) and Yordpratum et al. (2011) isolated phages from soil sample [28, 34]. This indicates that phages can be isolated from wide variety of sources. However, from our study, lytic phage against Gram-positive bacteria including* Staphylococcus *spp. was not isolated. Further studies need to be carried out to completely investigate the presence of efficient lytic phages against Gram-positive bacteria in Nepalese rivers.

Alongside, a total of sixty-seven phages were isolated from ten water samples using 8 different host organisms. Among them, forty-seven phages were selected for analyzing its host range which included phages against* Escherichia coli *(ΦEC)*, Klebsiella pneumoniae *(ΦKP),* Enterobacter *(ΦEB),* Serratia *(ΦSER),* Proteus *(ΦPRO)* and Salmonella *(ΦSAL). The result was quite similar to the study conducted by Duraisamy et al. (2015) from India, in which 46 bacteriophages were isolated against 20 different MDR and ESBL strains from hospital effluents. Among them some phages were named as Mm81, Ec84, Ps85, En833, Sal836, and Ec8ATCC against* Morganella morganii*,* Escherichia coli, Pseudomonas aeruginosa, Enterobacter cloacae, Salmonella *sp., and* E. coli* ATCC, respectively [35]. Likewise, Uchiyama et al. in 2008 isolated 30 phages using 16* Enterococcus faecalis *as a host [36]. Similarly, study carried by Carey-Smith et al. in 2006 isolated 8 phages from sewage using 3* Salmonella* serovars (*S. typhimurium* PT160,* Salmonella* LT2, and* S. infantis*) [14]. The study conducted by Kȩsik-Szeloch et al. (2013) demonstrated 32 lytic bacteriophages from 8 different water samples by using ESBL producing* K. pneumoniae* strains as host [19]. Similarly, in the study of Wu et al. (2007), twelve phages were isolated using a clinical strain* K. pneumoniae *6 and* K. pneumoniae *10693 as host cell by processing 254 hospital samples including catheter washings, patient specimens, and wastewater from drainages [37]. The isolation difference in these studies might be attributable to the variation in types of samples, geographic location, and host used in the study.

Another objective of this study was to determine the host range of isolated phages. Of all the 47 phages, ΦSER1 had broad host range with 85% effectiveness when tested against 20 different bacterial strains. This phage was able to lyse* Escherichia coli*,* Klebsiella pneumoniae*,* Enterobacter* spp.,* Pseudomonas *spp. including MDR, and* Citrobacter *spp. along with MBL and genetically characterized ESBL strains. However, in the literature, the effectiveness of* Serratia* phages is scarce. Like, Matshushita et al. (2009) showed that* Serratia* phages (KSP20, KSP90, and KSP100) could not lyse other species of* Enterobacteriaceae* (*Proteus vulgaris*,* Citrobacter freundii*,* Enterobacter cloacae*,* Hafnia alvei*,* Klebsiella pneumoniae*, and* Escherichia coli* strains). To date, screening of* Serratia* phage against MBL and ESBL strains similar to our study had not been documented.

In our study,* Klebsiella* phages (ΦKP6, ΦKP7, ΦKP8, and ΦKP9) were specific to only* Klebsiella pneumoniae *indicating its narrow host range. Similarly, the study carried by Chhibber et al. revealed that the phage SS specific to* K. pneumoniae* had narrow host range as only 7 out of 20 clinical isolates were sensitive to this phage [7]. Likewise, the study conducted by Hsu et al. showed that lytic phage (KN2) of the* K. pneumoniae* killed* K. pneumoniae* strains but did not cause lysis of other* Enterobacteriaceae*, including* Enterobacter aerogenes*,* Escherichia coli*, and* Salmonella typhimurium*. This result also suggested that phage was specific to* K. pneumoniae* [38]. The study of Volozhantsev et al. (2016) showed that the phage vB_KpnP_KpV289 lysed 15 out of 140 (10.7%)* K. pneumoniae* strains [39].

In the study carried out by Wu et al. (2007),* Klebsiella* phage (kpp95) was found effective against* Klebsiella oxytoca *(14 out of 14),* Enterobacter agglomerans *(7/10), and* Serratia marcescens (5/5),* ESBL strains of* K. pneumoniae *indicating its broad host range [37]. But the* Klebsiella* phages (ΦKP6, ΦKP7, ΦKP8, and ΦKP9) of our study did not reveal such type of effectiveness. This difference may be due to genetic diversity of phages and geographical distribution of bacterial isolates. Similarly, phages against* Escherichia, Enterobacter, Salmonella, *and* Proteus *were also found effective against few isolates. In addition, lytic phages from our study were also found effective against carbapenem resistant strains of* Enterobacteriaceae*. This represents the potential of phage utility in treating MDR infection caused by these bugs.

The result of this study suggests that bacteriophages have promising effect against clinical isolates of bacteria; hence its application can be a welcome addition in the treatment of antibiotic resistant pathogens. The limited number of bacterial strains in this study might be insufficient to conclude the host specificity of the phages. For successful therapeutic application, a group of broad host range or specific phages are required to infect potential pathogenic host strains involved in the outbreak of disease. Hence, multiple samples and more bacterial strains from same species or genera, particularly antibiotic resistant strains, should be included to determine host specificity. Further research including molecular analysis, full genome sequencing and clinical trial studies would be very much useful in the selection of phage type for phage therapy.

## 5. Conclusions

Isolation of potential phages lytic against various indicator organisms commonly involved in human infections is a major finding of this study. Notably, we found few phages lytic against multidrug-resistant pathogenic bacteria including ESBL and MBL producers. This promising effect against MDR pathogens has raised the probable utility of these phages for biological control of bacterial infection. Further characterization of specific phages is needed to explore the potential use of these phages for their clinical application.

## Figures and Tables

**Table 1:** Spectrum of phages against various pathogenic bacteria and their concentration.

River site	* Escherichia coli*		* Klebsiella *spp.		*Enterobacter *spp.		*Citrobacter *spp.		*Proteus *spp.		*Pseudomonas *spp.		*Serratia *spp.		*Salmonella *spp.	
	Effect	Pfu/ml	Effect	Pfu/ml	Effect	Pfu/ml	Effect	Pfu/ml	Effect	Pfu/ml	Effect	Pfu/ml	Effect	Pfu/ml	Effect	Pfu/ml
	(+/−)		(+/−)		(+/−)		(+/−)		(+/−)		(+/−)		(+/−)		(+/−)	
(1)	+	2.2 × 10^2^	+	1.5 × 10^3^	+	1.0 × 10^2^	+	0.63 × 10^2^	+	0.04 × 10^2^	−	—	+	2.0 × 10^2^	−	—
(2)	+	3.9 × 10^2^	+	3.3 × 10^3^	+	4.5 × 10^2^	+	0.70 × 10^2^	+	0.05 × 10^2^	−	—	+	3.1 × 10^2^	−	—
(3)	+	2.3 × 10^2^	+	4.0 × 10^3^	+	0.06 × 10^2^	+	0.55 × 10^2^	+	0.01 × 10^2^	+	0.06 × 10^2^	+	1.9 × 10^2^	−	—
(4)	+	2.1 × 10^2^	+	2.9 × 10^3^	+	2.5 × 10^2^	+	0.88 × 10^2^	+	0.01 × 10^2^	−	—	+	2.8 × 10^2^	−	—
(5)	+	2.5 × 10^2^	+	1.8 × 10^3^	+	1.4 × 10^2^	+	0.59 × 10^2^	+	0.09 × 10^2^	+	0.08 × 10^2^	+	0.45 × 10^2^	−	—
(6)	+	3.3 × 10^2^	+	2.0 × 10^3^	+	3.5 × 10^2^	+	0.77 × 10^2^	+	0.1 × 10^2^	+	0.05 × 10^2^	+	1.5 × 10^2^	+	0.21 × 10^2^
(7)	+	0.06 × 10^2^	+	1.5 × 10^3^	+	1.9 × 10^2^	+	0.33 × 10^2^	−	—	+	0.04 × 10^2^	+	1.7 × 10^2^	+	0.05 × 10^2^
(8)	+	2.6 × 10^2^	+	2.4 × 10^3^	+	2.2 × 10^2^	+	0.68 × 10^2^	+	0.04 × 10^2^	−	—	+	0.44 × 10^2^	+	0.19 × 10^2^
(9)	+	2.1 × 10^2^	+	2.5 × 10^3^	+	3.2 × 10^2^	+	0.75 × 10^2^	−	—	−	—	+	2.0 × 10^2^	−	—
(10)	+	2.5 × 10^2^	+	2.9 × 10^3^	+	1.8 × 10^2^	+	0.58 × 10^2^	+	0.05 × 10^2^	+	0.08 × 10^2^	+	0.4 × 10^2^	+	0.13 × 10^2^

(1) Rudramati river (Chabahil); (2) Bishnumati river (Teku); (3) Bagmati river (Sankhamool); (4) Bishnumati river (Soalteemode); (5) Bishnumati river (Balkhu); (6) Manohara river (Koteshwor); (7) Kirtipur river; (8) Bagmati river (Thapathali); (9) Rudramati river (Kalopool); (10) Hanumante river (Bhaktapur); [+ for effective, − for non effective phage].

**Table 2:** * Escherichia coli* phages (ΦEC) and their infectivity.

S. number	Isolates	ΦEC1	ΦEC2	ΦEC3	ΦEC4	ΦEC5	ΦEC6	ΦEC7	ΦEC8	ΦEC9	ΦEC10
(1)	*Escherichia coli*	+ + + +	+ + + +	+ +	+ + +	+ + +	+ +	+ +	+ +	+ +	++
(2)	*Klebsiella *spp.	+ + +	+ + +	+ +	+ + +	−	−	+ +	−	−	−
(3)	*Enterobacter *spp.	+	+	+	−	−	−	−	−	−	−
(4)	*Proteus *spp.	−	−	−	−	+ + +	−	−	−	−	+
(5)	*Pseudomonas *spp.	−	−	−	−	−	−	−	−	−	−
(6)	*Serratia *spp.	−	+	+	+	−	−	−	−	−	−
(7)	*Salmonella *spp.	−	−	−	−	−	−	−	−	−	−
(8)	*Citrobacter *spp.	+ + + +	+ + +	+ + + +	+ + +	+ +	+	−	−	−	−
(9)	ESBL1 (bla-SHV + bla-TEM)	+ + +	+ + +	−	+ + +	+ + + +	+	+	+	+	+
(10)	ESBL6 (bla-TEM)	+ +	+ +	+	+ +	+ + +	+ +	−	−	−	+
(11)	ESBL8 (bla-TEM)	+ +	+ +	+	+ +	+	+	+	+	+	+
(12)	ESBL13 (bla-TEM)	+ + + +	+ + + +	+ + + +	+ + + +	−	−	−	−	−	+
(13)	ESBL15 (bla-TEM)	+ + +	+ +	+ +	+ +	+	+	−	−	+	+
(14)	ESBL 22 (bla-CTX-M)	+ + +	+ + +	+ + +	+ + +	+ +	+	−	+	+	+
(15)	MBL1	−	−	−	−	−	−	−	−	−	−
(16)	MBL2	−	−	−	−	−	−	−	−	−	−
(17)	MBL5	−	−	−	−	−	−	−	−	−	−
(18)	MBL10	−	−	−	−	−	−	−	−	−	−
(19)	MBL34	−	−	−	−	−	−	−	−	−	−
(20)	MDR *Pseudomonas*	−	−	−	−	−	−	−	−	−	−

(+4 = very effective, +3 = fairly effective, +2 = moderately effective, +1 = slightly effective, (−) = not effective, ΦEC = phage against *Escherichia coli*.)

**Table 3:** * Klebsiella pneumoniae* phages (ΦKP) and their infectivity.

S. number	Isolates	ΦKP1	ΦKP2	ΦKP3	ΦKP4	ΦKP5	ΦKP6	ΦKP7	ΦKP8	ΦKP9	ΦKP10
(1)	*Escherichia coli *	+ +	+ +	+	+	−	−	−	−	−	−
(2)	*Klebsiella *spp.	+ + + +	+ + + +	+ + +	+ + +	+ +	+ + +	+ +	+ +	+ + +	+ + +
(3)	*Enterobacter *spp.	+ +	+ +	−	−	−	−	−	−	−	−
(4)	*Proteus *spp.	−	−	−	−	−	−	−	−	−	+ +
(5)	*Pseudomonas *spp.	−	−	−	−	−	−	−	−	−	−
(6)	*Serratia *spp.	−	−	−	−	−	−	−	−	−	−
(7)	*Salmonella *spp.	−	−	−	−	−	−	−	−	−	−
(8)	*Citrobacter *spp.	−	−	−	−	−	−	−	−	−	−
(9)	ESBL1 (bla SHV + bla TEM)	+	+	−	−	−	−	−	−	−	−
(10)	ESBL6 (bla-TEM)	+ +	−	−	−	+ +	−	−	−	−	−
(11)	ESBL8 (bla-TEM)	−	−	−	−	−	−	−	−	−	−
(12)	ESBL13 (bla-TEM)	−	−	−	−	−	−	−	−	−	−
(13)	ESBL15 (bla-TEM)	−	−	−	−	−	−	−	−	−	−
(14)	ESBL22 (bla-CTX-M)	−	−	−	−	−	−	−	−	−	−
(15)	MBL1	+ +	+	+	+	+	−	−	−	−	−
(16)	MBL2	−	−	−	−	−	−	−	−	−	−
(17)	MBL5	−	−	−	−	−	−	−	−	−	−
(18)	MBL10	−	−	−	−	−	−	−	−	−	−
(19)	MBL34	−	−	−	−	−	−	−	−	−	−
(20)	MDR *Pseudomonas*	−	−	−	−	−	−	−	−	−	−

**Table 4:** *Enterobacter *phages (ΦEB) and their infectivity.

S. number	Isolates	ΦEB1	ΦEB2	ΦEB3	ΦEB4	ΦEB5	ΦEB6	ΦEB7	ΦEB8	ΦEB9	ΦEB10
(1)	*Escherichia coli*	+ + +	+ +	+ + +	+ +	+	+	−	+	−	−
(2)	*Klebsiella *spp.	+ + +	+ +	+ + +	+ + +	+ +	+ +	+ +	+ +	+ +	−
(3)	*Enterobacter *spp.	+ +	+ +	+ + +	+ + +	+	+ + +	+ + + +	+ +	+	+ + +
(4)	*Proteus *spp.	−	−	−	−	−	−	−	−	−	−
(5)	*Pseudomonas *spp.	−	−	−	−	−	−	−	−	−	−
(6)	*Serratia *spp.	−	−	+	−	+	+	+	+	+	+
(7)	*Salmonella *spp.	−	−	−	−	+	−	+	+	+	+
(8)	*Citrobacter *spp.	+ + + +	+ + +	+	+	+	+	+	+	+	+
(9)	ESBL1 (bla-SHV + bla-TEM)	+	+ +	−	−	−	−	−	−	−	−
(10)	ESBL6 (bla-TEM)	−	+ + +	−	−	−	−	−	−	+ +	+
(11)	ESBL8 (bla-TEM)	+ +	+ +	−	−	+	+	−	−	−	+
(12)	ESBL13 (bla-TEM)	−	+ +	+ + +	+ + +	+	−	+ +	+	+ + +	+
(13)	ESBL15 (bla-TEM)	+ + + +	+ +	+	+	+	−	−	−	−	−
(14)	ESBL22 (bla-CTX-M)	+ + +	+ +	+ + +	+ + +	+	+	+	+	+	+
(15)	MBL1	−	−	−	−	−	−	−	−	−	−
(16)	MBL2	−	−	−	−	+	+	+	+	+	+
(17)	MBL5	−	−	−	−	−	−	−	−	−	−
(18)	MBL10	−	−	−	−	−	−	−	−	−	−
(19)	MBL34	−	−	−	−	−	−	−	−	−	−
(20)	MDR* Pseudomonas*	−	−	−	−	−	−	−	−	−	−

**Table 5:** *Serratia *phages (ΦSER) and their infectivity.

S. number	Isolates	ΦSER1	ΦSER2	ΦSER3	ΦSER4	ΦSER5	ΦSER6	ΦSER7	ΦSER8	ΦSER9	ΦSER10
(1)	*Escherichia coli*	+ + +	−	+ +	+ +	+	+ + +	−	−	−	−
(2)	*Klebsiella *spp.	+ + +	+ + +	+ + +	+ + + +	−	+ + + +	+ + +	+ + +	−	−
(3)	*Enterobacter *spp.	+ + +	−	+ +	+ + +	+ +	+ + +	+	+ + +	+ +	+ +
(4)	*Proteus *spp.	−	−	−	−	−	−	−	−	−	−
(5)	*Pseudomonas *spp.	+ + +	−	−	−	+ + + +	−	−	−	−	−
(6)	*Serratia *spp.	+ +	+ +	+ +	+	+ + + +	+ +	+ +	+ + + +	+ + +	+ +
(7)	*Salmonella *spp.	−	−	−	−	+ + + +	+	+	+ +	+ + +	+
(8)	*Citrobacter *spp.	+	−	−	−	−	−	−	−	−	−
(9)	ESBL1 (bla SHV + bla TEM)	+ + +	+	−	+	−	−	−	−	−	−
(10)	ESBL6 (bla-TEM)	−	+	−	−	−	−	−	−	−	−
(11)	ESBL8 (bla-TEM)	+ +	−	+	−	−	+	−	−	−	−
(12)	ESBL13 (bla-TEM)	+ + +	−	−	−	−	−	+	+	+	+
(13)	ESBL15 (bla-TEM)	+	−	+	+	−	−	−	−	−	−
(14)	ESBL22 (bla-CTX-M)	+	−	−	−	−	−	−	+ +	+ +	+ +
(15)	MBL1	+	−	−	−	−	−	−	−	−	−
(16)	MBL2	+	−	−	−	−	+	+	+	+	+
(17)	MBL5	+	−	−	−	−	−	−	−	−	−
(18)	MBL10	+	−	−	−	−	−	−	−	−	−
(19)	MBL34	+	−	−	−	−	−	−	−	−	−
(20)	MDR* Pseudomonas*	+ +	−	−	−	+ +	−	−	−	−	−

**Table 6:** *Salmonella *(ΦSAL)* and Proteus* (ΦPRO) phages and their infectivity.

S. N	Isolates	ΦPRO5	ΦPRO6	ΦPRO7	ΦPRO10	ΦSAL6	ΦSAL8	ΦSAL10
(1)	*Escherichia coli*	−	−	−	−	−	−	−
(2)	*Klebsiella * spp.	+	+ + +	+ + +	+	−	+ + +	+ + +
(3)	*Enterobacter * spp.	−	+ + +	+	−	+ +	−	−
(4)	*Proteus spp*	+ + + +	+ + + +	+ + +	+ + +	−	−	−
(5)	*Pseudomonas spp*	−	−	−	−	−	−	−
(6)	*Serratia spp*	+	+	−	−	+	+	+ + +
(7)	*Salmonella spp*	−	−	+	−	+ + + +	+ +	+ + +
(8)	*Citrobacter spp*	−	−	−	−	−	−	−
(9)	ESBL1 (bla-SHV + bla-TEM)	−	−	−	−	+	+	+
(10)	ESBL6 (bla-TEM)	−	−	−	−	+	+	+ + + +
(11)	ESBL8 (bla-TEM)	−	−	−	−	+	+	+
(12)	ESBL13 (bla-TEM)	−	+ +	−	−	+	+	+ +
(13)	ESBL15 (bla-TEM)	−	−	−	−	−	−	−
(14)	ESBL 22 (bla-CTXM)	−	+ + +	−	+ + +	+ + + +	−	+
(15)	MBL1	−	−	−	−	+	+	+ + + +
(16)	MBL2	−	+	−	−	+	−	−
(17)	MBL5	−	−	−	−	−	−	−
(18)	MBL10	−	−	−	−	−	−	−
(19)	MBL34	−	−	−	−	−	−	−
(20)	MDR* Pseudomonas*	−	−	−	−	−	−	−

## Abbreviations

CTX-M: Cefotaxime-Munich
ESBL: Extended Spectrum *β*-Lactamases
*E. coli*: * Escherichia coli*
ICTV: International Committee for Taxonomy of Viruses
MDR: Multidrug-Resistant
MBL: Metallo b-Lactamases
PFU: Plaque forming unit
PCR: Polymerase Chain Reaction
SHV: Sulfhydryl variable
TEM: Temoneira.

## References

[1] Kutateladze M., Adamia R. (2010). Bacteriophages as potential new therapeutics to replace or supplement antibiotics. *Trends in Biotechnology*.

[2] Yu Y.-P., Gong T., Jost G., Liu W.-H., Ye D.-Z., Luo Z.-H. (2013). Isolation and characterization of five lytic bacteriophages infecting a Vibrio strain closely related to Vibrio owensii. *FEMS Microbiology Letters*.

[3] Hanlon G. W. (2007). Bacteriophages: an appraisal of their role in the treatment of bacterial infections. *International Journal of Antimicrobial Agents*.

[4] Ahn J., Biswas D. (2014). Influence of bacteriophage P22 on the inflammatory mediator gene expression in chicken macrophage HD11 cells infected with Salmonella Typhimurium. *FEMS Microbiology Letters*.

[5] Donovan D. M., Lardeo M., Foster-Frey J. (2006). Lysis of staphylococcal mastitis pathogens by bacteriophage phi11 endolysin. *FEMS Microbiology Letters*.

[6] De vos D., Pirnay JP. (2015). Phage therapy: could viruses help resolve the worldwide antibiotic crisis?. *AMR Control 2015; Overcoming Global Antibiotic Resistance*.

[7] Chhibber S., Kaur S., Kumari S. (2008). Therapeutic potential of bacteriophage in treating *Klebsiella pneumoniae* B5055-mediated lobar pneumonia in mice. *Journal of Medical Microbiology*.

[8] Międzybrodzki R., Borysowski J., Weber-Dąbrowska B. (2012). Clinical aspects of phage therapy. *Advances in Virus Research*.

[9] Loc-Carrillo C., Abedon S. T. (2011). Pros and cons of phage therapy. *Bacteriophage*.

[10] Mihu M. R., Martinez L. R. (2011). Novel therapies for treatment of multi-drug resistant Acinetobacter baumannii skin infections.. *Virulence*.

[11] Bolocan A. S., Callanan J., Forde A., Ross P., Hill C. (2016). Phage therapy targeting Escherichia coli-a story with no end?. *FEMS Microbiology Letters*.

[12] Jin J., Li Z.-J., Wang S.-W. (2012). Isolation and characterization of ZZ1, a novel lytic phage that infects Acinetobacter baumannii clinical isolates. *BMC Microbiology*.

[13] Al-Fendi A., Shueb R. H., Ravichandran M., Yean C. Y. (2014). Isolation and characterization of lytic vibriophage against Vibrio cholerae O1 from environmental water samples in Kelantan, Malaysia. *Journal of Basic Microbiology*.

[14] Carey-Smith G. V., Billington C., Cornelius A. J., Hudson J. A., Heinemann J. A. (2006). Isolation and characterization of bacteriophages infecting *Salmonella* spp. *FEMS Microbiology Letters*.

[15] Matsushita K., Uchiyama J., Kato S.-I. (2009). Morphological and genetic analysis of three bacteriophages of Serratia marcescens isolated from environmental water: Research Letter. *FEMS Microbiology Letters*.

[16] Wang J., Hu B., Xu M. (2006). Therapeutic effectiveness of bacteriophages in the rescue of mice with extended spectrum *β*-lactamase-producing Escherichia coli bacteremia. *International Journal of Molecular Medicine*.

[17] Mann N. H. (2008). The potential of phages to prevent MRSA infections. *Research in Microbiology*.

[18] Elbreki M., Ross R. P., Hill C., O'Mahony J., McAuliffe O., Coffey A. (2014). Bacteriophages and Their Derivatives as Biotherapeutic Agents in Disease Prevention and Treatment. *Journal of Viruses*.

[19] Kęsik-Szeloch A., Drulis-Kawa Z., Weber-Dąbrowska B. (2013). Characterising the biology of novel lytic bacteriophages infecting multidrug resistant Klebsiella pneumoniae. *Virology Journal*.

[20] Debattista J. (2004). Phage therapy: Where East meets West. *Expert Review of Anti-infective Therapy*.

[21] Viertel T. M., Ritter K., Horz H.-P. (2014). Viruses versus bacteria-novel approaches to phage therapy as a tool against multidrug-resistant pathogens. *Journal of Antimicrobial Chemotherapy*.

[22] Isenberg HD. (2004). *Clinical Microbiology procedure handbook*.

[23] (2012). *Performance Standards for Antimicrobial Disk Susceptibility Tests*.

[24] Magiorakos A.-P., Srinivasan A., Carey R. B. (2012). Multidrug-resistant, extensively drug-resistant and pandrug-resistant bacteria: an international expert proposal for interim standard definitions for acquired resistance. *Clinical Microbiology and Infection*.

[25] Parajuli N. P., Maharjan P., Joshi G., Khanal P. R. (2016). Emerging perils of extended spectrum *β* -lactamase producing enterobacteriaceae clinical isolates in a teaching hospital of Nepal. *BioMed Research International*.

[26] Yong D., Lee K., Yum J. H., Shin H. B., Rossolini G. M., Chong Y. (2002). Imipenem-EDTA disk method for differentiation of metallo-*β*-lactamase-producing clinical isolates of *Pseudomonas* spp. and *Acinetobacter* spp.. *Journal of Clinical Microbiology*.

[27] Vitaliano J., Fromm S., Packer D., Reid R., Pikanowski R. (2007). Recovery of benthic macrofauna from sewage sludge disposal in the New York Bight. *Marine Ecology Progress Series*.

[28] Seaman P. F., Day M. J. (2007). Isolation and characterization of a bacteriophage with an unusually large genome from the Great Salt Plains National Wildlife Refuge, Oklahoma, USA. *FEMS Microbiology Ecology*.

[29] Yang H., Liang L., Lin S., Jia S. (2010). Isolation and characterization of a virulent bacteriophage AB1 of Acinetobacter baumannii. *BMC Microbiology*.

[30] Mulani MS., Azhar S., Azharuddin S., Tambe S. (2015). Harnessing the Power of Bacteriophage for Pathogen Reduction in Wastewater. *Int J Curr Microbiol App Sci*.

[31] Shukla S. K., Hirpurkar S., Singh S. K., Rajoria R. (2014). Isolation of phage from animal waste of different LSF and their utility in phage therapy. *International Journal of Current Microbiology and Applied Sciences*.

[32] Li L., Zhang Z. (2014). Isolation and characterization of a virulent bacteriophage SPW specific for Staphylococcus aureus isolated from bovine mastitis of lactating dairy cattle. *Molecular Biology Reports*.

[33] Alonso M. D. C., Rodríguez J., Borrego J. J. (2002). Characterization of marine bacteriophages isolated from the Alboran Sea (Western Mediterranean). *Journal of Plankton Research*.

[34] Yordpratum U., Tattawasart U., Wongratanacheewin S., Sermswan R. W. (2011). Novel lytic bacteriophages from soil that lyse Burkholderia pseudomallei. *FEMS Microbiology Letters*.

[35] Duraisamy N., Nachimuthu R., Vaithilingam K., Pandiyan R., Ebenezer K. S., Velu R. K. (2015). Distribution, Isolation and Characterization of Lytic Bacteriophages against MDR and ESBL Producing Pathogens from Hospital Effluents. *Asian Journal of Pharmaceutical and Clinical Research*.

[36] Uchiyama J., Rashel M., Maeda Y. (2008). Isolation and characterization of a novel *Enterococcus faecalis* bacteriophage *φ*EF24C as a therapeutic candidate. *FEMS Microbiology Letters*.

[37] Wu L.-T., Chang S.-Y., Yen M.-R., Yang T.-C., Tseng Y.-H. (2007). Characterization of extended-host-range pseudo-T-even bacteriophage Kpp95 isolated on Klebsiella pneumoniae. *Applied and Environmental Microbiology*.

[38] Hsu C.-R., Lin T.-L., Pan Y.-J., Hsieh P.-F., Wang J.-T. (2013). Isolation of a Bacteriophage Specific for a New Capsular Type of Klebsiella pneumoniae and Characterization of Its Polysaccharide Depolymerase. *PLoS ONE*.

[39] Volozhantsev N. V., Myakinina V. P., Popova A. V. (2016). Complete genome sequence of novel T7-like virus vB_KpnP_KpV289 with lytic activity against Klebsiella pneumoniae. *Archives of Virology*.

